# External validation of brain arteriovenous malformation haemorrhage scores, AVICH, ICH and R2eD

**DOI:** 10.1007/s00701-022-05190-1

**Published:** 2022-04-18

**Authors:** Basel A. Taweel, Conor S. Gillespie, George E. Richardson, Mohammad A. Mustafa, Tamara Ali, Abdurrahman I. Islim, Cathal J. Hannan, Emmanuel Chavredakis

**Affiliations:** 1 Institute of Systems, Integrative and Molecular Biology (ISMIB), University of Liverpool, Liverpool, UK; 2 The Walton Centre NHS Foundation Trust, Liverpool, UK; 3 Clinical Sciences Centre, Aintree University Hospital, Fazakerley, Longmoor Lane, Liverpool, L9 7AL UK

**Keywords:** Arteriovenous malformations, Cerebral haemorrhage, Prognosis, Cerebrovascular disorders

## Abstract

**Purpose:**

To externally validate the arteriovenous malformation-related intracerebral haemorrhage (AVICH), intracerebral haemorrhage (ICH), and novel haemorrhage presentation risk score (R2eD) in brain arteriovenous malformations.

**Methods:**

Adult patients diagnosed radiologically with an arteriovenous malformation (AVM) at a tertiary neurosurgical centre between 2007 and 2018 were eligible for inclusion. Both the AVICH and ICH scores were calculated for AVM-related symptomatic haemorrhage (SH) and compared against the modified Rankin scale (mRS) at discharge and last follow-up, with unfavourable outcome defined as mRS > 2. R2eD scores were stratified based on presentation with SH. External validity was assessed using Harrel’s C-statistic.

**Results:**

Two hundred fifty patients were included. Mean age at diagnosis was 46.2 years [SD = 16.5]). Eighty-seven patients (34.8%) had a SH, with 83 included in the analysis. Unfavourable mRS outcome was seen in 18 (21.6%) patients at discharge and 18 (21.6%) patients at last follow-up. The AVICH score C-statistic was 0.67 (95% confidence interval [CI], 0.53–0.80) at discharge and 0.70 (95% CI, 0.56–0.84) at last follow-up. The ICH score C-statistic was 0.78 (95% CI 0.67–0.88), at discharge and 0.80 (95% CI 0.69–0.91) at last follow-up. The R2eD score C-statistic for predicting AVM haemorrhage was 0.60 (95% CI, 0.53–0.67).

**Conclusions:**

The AVICH score showed fair-poor performance, while the ICH score showed good-fair performance. The R2eD score demonstrated poor performance, and its clinical utility in predicting AVM haemorrhage remains unclear.

## Introduction

Brain arteriovenous malformations (AVMs) are vascular lesions characterised by arteriovenous shunting via a central entanglement of arterial and venous components known as a nidus. These lesions are thought to be congenital and carry a lifetime risk of haemorrhage, with an estimated annual haemorrhage rate of 2–4% [5, 12].

Haemorrhage is the most common clinical presentation of AVMs (~ 50%), with AVM haemorrhage representing 2–4% of all haemorrhagic strokes and 1–2% of all strokes [1].

Treatment modalities for AVM include microsurgical resection [25], stereotactic radiosurgery and endovascular therapy, as well as conservative management [18]. In addition to the risk of haemorrhage represented by the natural history AVMs, each modality of interventional therapy for AVMs carries a risk of neurological morbidity [6, 16], thus careful selection of lesions for interventional therapy is paramount.

Currently, no established paradigm exists to identify lesions that are likely to cause disability later in life, though clinical models have been proposed that attempt this. The R2eD score [10] stratifies AVMs based on both lesion and patient characteristics to ascertain those at risk of haemorrhage (Table 1). The original intracerebral haemorrhage (ICH) score [13] has been suggested as a predictor of disability following AVM ICH [3]. The AVM-related ICH (AVICH) score [20] is a score based on the original ICH score, which was developed in an attempt to more accurately predict the occurrence of neurological disability following AVM-related ICH (Table 2).

**Table 1:** R2ED score components and component definitions

Component	Definition	R2eD
Race	White	0
	Non-white	2
Location	Other location (not deep)	0
	Deep	1
AVM size	Large ≥ 30 mm	0
	Small < 30 mm	1
Deep venous drainage	No	0
	Yes	1
Monoarterial feeding	No	0
	Yes	1
Score range		0–6

*R2eD* novel haemorrhage presentation risk score

**Table 2:** List of the components of the AVICH and ICH scores

Component	Definition	AVICH score	ICH score
Nidus size	< 3	1	/
	3–6	2	/
	> 6	3	/
Deep venous drainage	No	0	/
	Yes	1	/
Eloquence	No	0	/
	Yes	1	/
Age (AVICH thresholds)	< 20 years	1	/
	20–40 years	2	/
	> 40 years	3	/
Age (ICH thresholds)	< 80 years	/	0
	≥ 80 years	/	1
Diffuse nidus	No	0	/
	Yes	1	/
GCS	3–4	0	0
	5–12	1	1
	13–15	2	2
ICH volume	< 30	0	0
	≥ 30	1	1
Intraventricular Haemorrhage	No	0	0
	Yes	1	1
Infratentorial Haemorrhage	No	/	0
	Yes	/	1
Score range		2–13	0–6

*AVICH* arteriovenous malformation-related intracerebral haemorrhage, *ICH* Intracerebral haemorrhage, *GCS* Glasgow coma scale

These clinical tools may have the potential to guide management of AVMs, by identifying high-risk lesions that are more likely to bleed and cause neurological disability due to haemorrhage.

This study aims to externally validate these tools using a mixed cohort and to assess their clinical utility.

## Methods

### Study design and patient population

Data for adult patients (> 18 years) diagnosed with AVMs at a single tertiary neuroscience centre between 2007 and 2018 was retrospectively collected. For inclusion, AVMs must have been confirmed by magnetic resonance imaging (MRI) or digital subtraction angiography (DSA). AVMs that were radiologically identified on computerised tomography scans with angiography (CTA) only, with no further MRI/DSA radiological confirmation were excluded, as a detailed study of certain lesion characteristics required for calculation of both the R2ED and AVICH scores (deep drainage, single feeder, diffuse nidus) could not be reliably assessed. The transparent reporting of a multivariable prediction model for individual prognosis or diagnosis (TRIPOD) statement was used to report the study findings [9]. The data that support the findings of this study are available from the corresponding author upon reasonable request.

### Baseline clinical and radiological characteristics

Data was collected from electronic patient notes and available imaging. AVM-specific data was collected in accordance with published consensus definitions [23]. The ABC/2 formula [15] was used to determine the volume of the ICH, and the Glasgow Coma Scale on admission was used to determine GCS for the purposes of calculating ICH and AVICH scores. Ethnicity data (required for calculating the R2eD score for AVM patients) was patient reported and was collected from patient records.

### Outcomes assessed

The primary outcome for the R2ED score was presentation with an ICH, either at first presentation, or during follow-up. This was conducted with the assumption that the risk of rupture of monitored lesions is identical to that of unknown lesions. Haemorrhages caused by AVM-related flow aneurysms were excluded from analysis. The outcomes for the ICH and AVICH score were modified Rankin scale (mRS) at discharge and last follow-up. Unfavourable outcome for the ICH and AVICH score is defined as mRS > 2. Patient outcomes were identified using electronic patient notes and clinical records.

### Statistical analysis

Baseline patient variables found to be significant in scoring models were compared between the original reported patient populations and between our patient cohorts. We assessed whether the individual values of continuous baseline variables lied within the reported ranges of the original study reporting development of the score. Any patient with continuous baseline characteristics lying outside of these ranges was excluded from the analysis.

Binary logistic regression was used to construct a generalised linear model for the purposes of assessing calibration. Regression was performed in accordance with the respective associated outcome (R2eD = occurrence of symptomatic haemorrhage (SH), AVICH and ICH = modified Rankin scale (mRS) > 2). For AVICH and ICH, both mRS after discharge, and at last follow-up were modelled, representing an assessment of the ability of scores to predict patient disability and dependence both short-term, and long-term. Date of last follow-up was determined to be the date of the most recent physical encounter with any clinician at our tertiary centre. Unique fitted values were ranked to model score outcomes as a continuous variable, for the purposes of constructing calibration plots. The resultant plots were used to visualise concordance of score prediction with observed outcome. Receiver operator curves (ROC) curves were plotted and C-statistics were used to assess discriminatory capability for each score. C-statistics are calculated by measuring the area under receiver operator curves (AUROC). The following grading scale was used to summarise the discriminatory capability of each model based on C-statistic: (1–0.9: excellent, 0.8–0.9: good, 0.7–0.8: fair, 0.6–0.7: poor, 0.5–0.6: very poor) (Table 5). R version 4.0.3 (used packages: pROC v.17.0.1, ggplot 3.3.3) was used for all the statistical analyses.

## Results

### Patient baseline characteristics

Patient baseline characteristics are reported in Table 3. Two hundred fifty patients with AVM (mean age = 46.24 [SD = 16.5], *M*:*F* = 0.92) were identified. The median duration of follow-up following haemorrhage was 33.74 months. No included patients had any co-morbid cavernous malformations, dural arteriovenous fistulas or hemangioblastomas. Two patients were found to have 2 AVMs each. As distinct nidi could be identified, these were deemed as separate lesions for the purposes of this study. Ranges of continuous variables of the original studies were found to encompass our reported ranges, with no patients excluded due to outlying characteristics.

**Table 3:** Baseline characteristics of the validation cohort

	All patients (*n* = 250) (%)	Haemorrhage pts (n = 83) (%)	Unfavourable outcome patients (*n* = 18) (%)
Age (years)	48.0	45.7	54.0
Male	120 (48.0)	46 (55.4)	8 (44.4)
Female	130 (52.0)	37 (44.6)	10 (55.4)
GCS			
13–15	-	60 (72.2)	9 (44.4)
5–12	-	15 (18.0)	5 (27.8)
3–4	-	8 (0.10)	4 (22.2)
Median ICH volume (cm^3)^	-	7.4	10.22
IVH	-	30 (36.1)	10 (55.6)
Infratentorial location	-	30 (36.1)	11 (61.1)
Ethnicity			
White	229 (91.6)	74 (89.2)	16 (88.9)
Non-white	21 (8.4)	9 (10.8)	2 (11.1)
Single feeder		21 (25.3)	2 (11.1)
Small size nidus (< 3 cm)	175 (70.0)	63 (75.9)	14 (77.8)
Large size nidus (≥ 3 cm)	75 (30.0)	20 (24.1)	4 (22.2)
Deep drainage	117 (46.8)	43 (51.8)	11 (61.1)
Deep location	69 (27.6)	30 (36.1)	11 (61.1)
Eloquent location	112 (44.8)	38 (45.8)	11 (61.1)
Diffuse nidus	66 (26.4)	30 (36.1)	7 (38.9)
Spetzler-Martin grade			
I	67 (26.8)	19 (22.9)	4 (22.2)
II	91 (36.4)	36 (43.4)	5 (27.8)
III	56 (22.4)	14 (16.9)	5 (27.8)
IV	29 (11.6)	13 (15.7)	4 (22.2)
V	7 (2.8)	1 (1.2)	0 (0.0)

*GCS* Glasgow coma scale, *ICH*: intracerebral haemorrhage, IVH intraventricular haemorrhage

### Patient outcomes

Ninety-three patients (37.2%) presented with SH. After exclusion of 6 flow-aneurysm haemorrhages, 87 patients remained, with a further 4 patients being excluded due to missing data. Of the included 83 haemorrhages, 74 (89.2%) presented with haemorrhage as the first presentation of their AVM, while 9 (10.8%) AVM haemorrhages were haemorrhages of known, conservatively managed lesions. Eighteen (21.7%) patients were found to have unfavourable mRS (> 2) at discharge, and 18 (21.7%) patients were found to have unfavourable mRS at last follow-up.

### External validation results

**Fig. 1:**
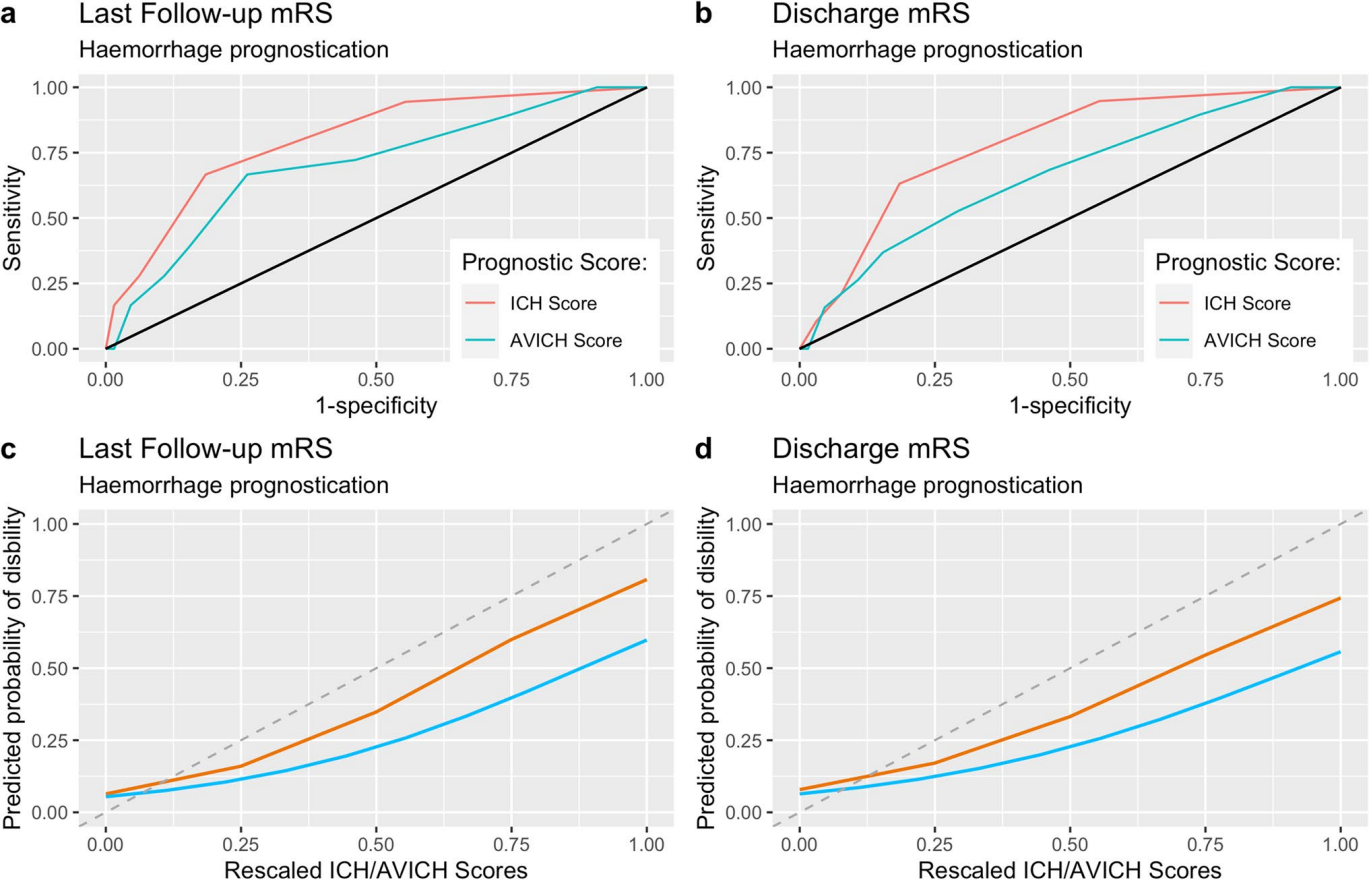
ROC curves and calibration plots used to assess discriminatory capability and model calibration for the ICH score and the AVICH score. Panels (a) and (c) show the ROC curves and calibration plots, respectively, for both scores using mRS at last follow-up as the outcome. (b) and (d) utilise mRS at discharge as the outcome of interest

**Fig. 2:**
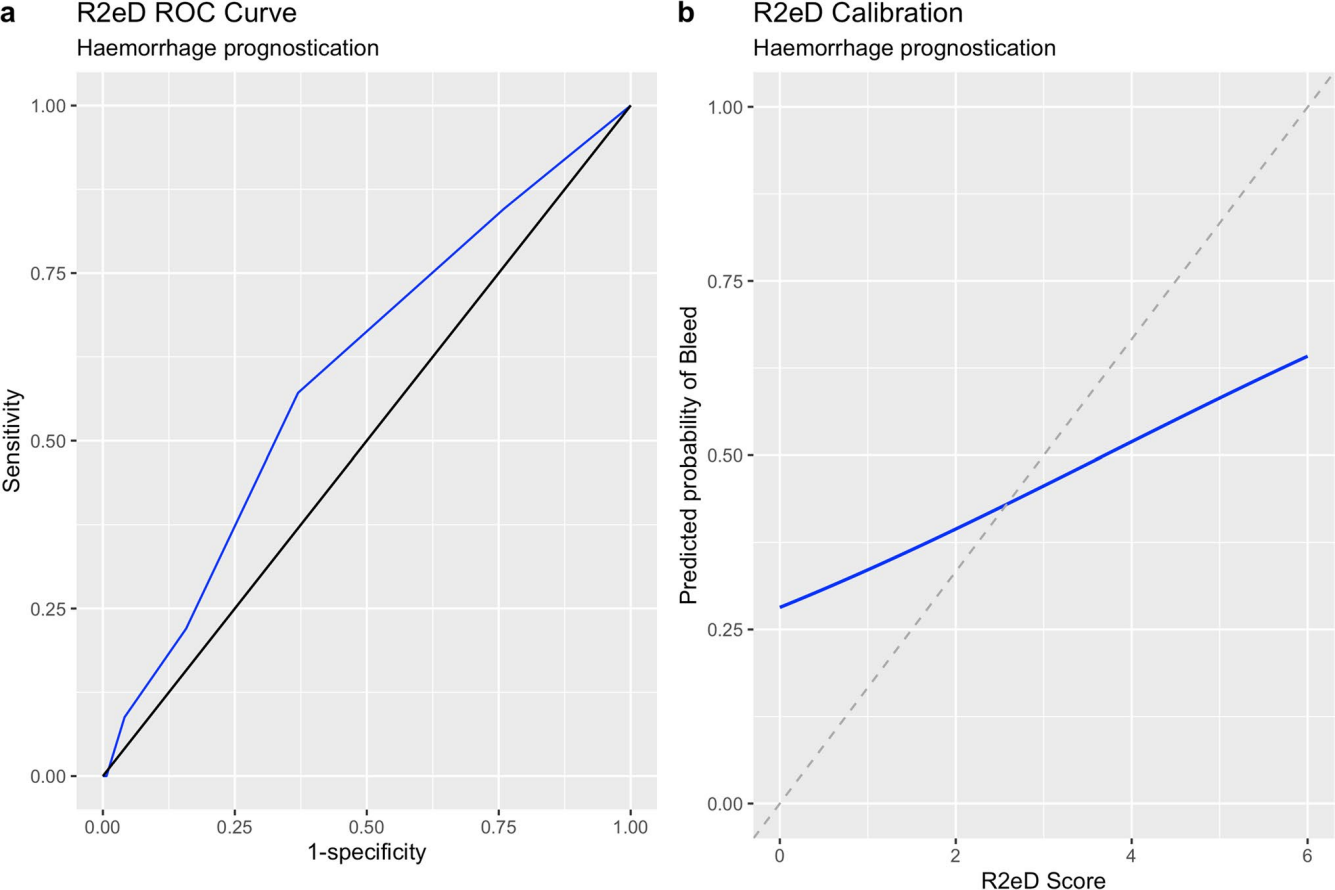
ROC curves (a) and calibration plots (b) used to assess discriminatory capability and model calibration for the R_2_eD score

The resulting ROC curves and calibration plots for each score are visualised in Figs. 1 and 2. The ICH score produced a C-statistic of 0.78 (95% CI 0.67–0.88) when predicting mRS at discharge, and a C-statistic of 0.80 (95% CI 0.69–0.91) when predicting mRS at last follow-up. The AVICH score produced a C-statistic of 0.67 (CI, 0.53–0.80) using discharge mRS and 0.70 (CI 0.56–0.84) using mRS at last follow-up.

The R2eD score produced a C-statistic of 0.60 (0.53–0.67) (Table 4). The calibration curve produced for R2eD showed a disparity between predicted and observed results. The ROC curve and calibration curve for the R2eD score are shown in Fig. 2.

**Table 4:** External validation results, showing the area under curve for each score. For the ICH and AVICH scores, discharge and follow-up mRS are used as short-term and longer-term outcomes, respectively

Score name	Discharge mRS AUC (95% CI)	Follow-up mRS AUC
ICH score	0.78 (0.67–0.88)	0.80 (0.69–0.91)
AVICH score	0.67 (0.53–0.80)	0.70 (0.56–0.84)
**Score name**	**Symptomatic haemorrhage AUC (95% CI)**	
R2eD score	0.60 (0.53–0.67)	

*mRS* modified Rankin scale, *AUC* area under curve, *CI* confidence intervals, *ICH* intracerebral haemorrhage, *AVICH* arteriovenous malformation-related intracerebral haemorrhage, *R2eD* novel haemorrhage presentation risk score

Using our aforementioned scale, the ICH score is found to be a good-fair predictor of disability following AVM rupture, while the AVICH score is found to be a fair-poor predictor. The R2eD score (AUC = 0.60) is found to be a poor and near-non-discriminatory predictor of AVM haemorrhage in our cohort (Table 5).

**Table 5:** The grading scale used to assess Harrel’s C statistic

Grade	AUC score
Excellent	1–0.9
Good	0.9–0.8
Fair	0.8–0.7
Poor	0.7–0.6
Non-discriminatory	0.6–0.5

AUC area under curve

## Discussion

Here we present the largest cohort validation of the R2eD score, in addition to an external validation of the ICH and AVICH scores for AVM rupture. We find that the ICH score is a good-fair predictor of disability following AVM-ICH and that the AVICH score is a fair-poor predictor of disability. Finally, we find that the R2ED score is a poor predictor of AVM haemorrhage in our cohort (Table 5).

Prior to this study, the R2eD score has been externally validated in a single cohort study of 122 patients [4] showing fair performance as a predictor of haemorrhage (AUC = 0.71); our study therefore represents the largest cohort validation of the R2eD to date.

The AVICH score has been externally validated by the developers of the score in an international multi-centre validation study [19], showing results surpassing that of the ICH score with an AUC of 0.77 for the AVICH score and AUC of 0.71 for the ICH score. Our study is the first independent assessment of the AVICH score in AVM haemorrhage. The ICH score has also been validated externally as a predictor of disability following AVM rupture [3], producing an AUC score of 0.89, with no comparison to the AVICH score.

The differences in the reported discriminatory power of haemorrhage scores between the multi-centre study and others may be explained by smaller event numbers, with only 18 incidences of unfavourable outcome in our cohort, 15 incidences in the initial ICH validation study, and 115 incidences of poor outcome in the multi-centre study. It should also be noted that our validation of the ICH score and the AVICH produced wide intersecting confidence intervals preventing definitive determination of a preferred score based on the discriminative capability reported in our study. For all scores, the AUC values produced were less than that of the original study reporting development of the score, which is expected when validating in an external cohort distinct from the original training cohort [8, 24].

### Clinical utility and implications — R2ED score

Grading scales such as the R2ED score are developed with the ultimate purpose of predicting future events, allowing clinicians to use the score to guide intervention in patients with AVM. The R2ED score was developed with retrospective data assuming that the natural history of all AVMs is similar to lesions that undergo conservative management. In this study, (which is also based on retrospective data,) we utilise that same assumption for the purposes of external validation of the R2ED score. We add to this, by including patients with conservatively managed AVMs that were discovered either incidentally or via other non-haemorrhagic symptomatic manifestation. While this technically leads to the inclusion of a cohort of patients that differs from the original training set (known vs. unknown lesions), this inclusion has the advantage of validating the R2ED score in a cohort that is more representative of the ideal clinical use case of the model. As noted by the developers of the R2ED score, the model was constructed based upon the study of characteristics that are observable in lesions that presented with haemorrhage. This does not, however, mean that these same characteristics are observable in high-risk AVMs that have not yet undergone haemorrhage. These issues arise from the use of retrospective data, and thus further prospective studies are required to determine the true clinical utility of this model and any future model that aims to risk-stratify AVMs. Finally, while the R2ED score aims to be utilised to predict haemorrhage, it does not address lesions that have previously bled, which have been found to have a significantly higher risk of subsequent haemorrhage [11, 22, 27].

### Clinical utility and implications—– ICH and AVICH scores

For the purposes of establishing a preferred model for predicting patient outcome following AVM rupture, model practicality must be taken into consideration. The ICH score is a model that was proposed in 2001 to assess mortality in all patients presenting with ICH and has been validated extensively for both its original use [7, 14, 21], and for use specifically to predict disability in AVM-ICH presentations [3]. The AVICH score is a bespoke score developed and validated for the purposes of predicting disability following AVM rupture. The use of the AVICH score therefore stipulates that a patient must have a previously known lesion that has bled, or that the lesion must be diagnosed at presentation in order for the model to be applicable. In our cohort, only 9 (10.8%) of our 83 studied bleeds were haemorrhages of known lesions, with the vast majority of AVM haemorrhagic presentations (89.2%) involving previously unknown AVMs. Furthermore, diagnosis of AVMs in the acute setting can prove difficult, as any underlying lesions can be obscured by the presenting haematoma on radiological investigations [2]. Additionally, the obscuring haematoma may impede the study of certain lesion characteristics necessary for use of the score, such as size, deep drainage and diffuseness of the lesion. These factors may obstruct use of the AVICH score in the acute setting to predict patient disability, and thus may preclude the use of AVICH in acute setting decision-making for AVM patients. The ICH score, however, is not subject to this limitation as it is useful both as a predictor of mortality in non-AVM-ICHs and as a predictor of disability in AVM-ICH. Furthermore, the ICH score does not rely on detailed radiological study of AVM characteristics and can be used based on radiological study of the presenting haematoma alone. The ICH score may therefore be applied in all ICH scenarios, irrespective of cause.

### Heterogeneity of arteriovenous malformations

Difficulties may arise in constructing models to predict clinical outcomes in AVMs, chief among which is the wide variability observed in the characteristics of different lesions. The developers of the R2eD score, reported the results of a literature review [10] which aimed to identify all studies of haemorrhage predictors in AVM, including AVM size, deep venous drainage, deep location, the number of feeding artery vessels, venous stenosis, patient ethnicity, and the presence of AVM-associated aneurysms. Wide variability was observed in the results of the identified studies, with some studies reporting the studied characteristic as a significant predictor of haemorrhage, and others reporting the converse. A recent study [26] demonstrated an attempt to utilise machine learning algorithms to construct predictive models in AVMs. The authors reported the produced models as unstable and uncertain. These studies and others [1, 17] indicate that AVM heterogeneity may obstruct the development of a haemorrhage prediction algorithm that encompasses all AVMs. These studies, when considered alongside our findings with relation to the R2eD score, may endorse further sub-setting of AVMs with the aim of finding more consistent patterns amongst similar lesions.

### Limitations of study

Our study has a number of limitations. It is a single-centre study based on retrospective patient data. Our cohort had 83 events of AVM haemorrhage for external validation of the R2eD score and 18 events of unfavourable neurological outcome for assessing the ICH and AVICH score, both of which lie under the recommended value of 100 events for validation studies [8]. Additionally, while the ICH score was found to have an AUC value greater than the AUC value for the AVICH score, the low number of events produced wide intersecting confidence intervals meaning that a preferred score cannot be definitively determined.

While the R2eD score showed poor performance in our study, it should be noted that the R2eD score includes ethnicity as a significant predictor of AVM haemorrhage, and our cohort was 91.6% white, which may not effectively illustrate the clinical utility of the R2eD score in more diverse populations.

## Conclusion

We present an external validation of three prognostic scores, the ICH, AVICH and R2eD scores that collectively aim to both predict and prognosticate outcome in AVM haemorrhage. We find that the ICH score is a good-fair predictor of disability following AVM haemorrhage, whereas the AVICH score is a fair-poor predictor. We find that the R2eD score is a poor predictor of AVM haemorrhage, and we recommend further prospective studies to help determine the true clinical utility of these models. Finally, we consider the possibility of an AVM categorisation system as a step to improve the predictive power of future scores that aim to predict AVM haemorrhage.
